# Varicella Pneumonia with Deep Venous Thrombosis and Pulmonary Embolism as Rare Presentations for Male: Case Report

**DOI:** 10.1055/s-0045-1802594

**Published:** 2025-04-02

**Authors:** Mohammad Dallah, Ahmad Hmaideh, Yaser Hashoom, Ammaar Kaesom, Nusima Gazhal

**Affiliations:** 1 Department of Pulmonology, Syrian Board of Medical Specialties, Idlib, Syria; 2 Department of Internal Medicine, Syrian Board of Medical Specialties, Idlib, Syria; 3 Syrian Public Health Network, Syria; 4 Department of Cardiology, Syrian Board of Medical Specialties, Idlib, Syria

**Keywords:** varicella pneumonia, deep venous thrombosis, pulmonary embolism, case report

## Abstract

This case report presents the case of a middle-aged man who experienced a rare and severe presentation of varicella infection, with the development of varicella pneumonia and the rare complications of deep venous thrombosis, and a subsequent pulmonary embolism. Despite being immunocompetent, the patient's varicella infection resulted in an atypical and complex clinical course. The individual initially presented with a vesicopustular rash, fever, and fatigue, which progressed to severe hypoxia and dyspnea. Radiological findings revealed diffuse bilateral nodular consolidation consistent with varicella pneumonia. Although varicella pneumonia is well documented, its occurrence in immunocompetent individuals is uncommon. In the context of varicella infection, it is very rare, particularly in the absence of immunodeficiency and with no other risk factors, in a male patient. This case underscores the importance of maintaining a high index of clinical suspicion to promptly diagnose and treat potentially life-threatening complications associated with varicella infection in otherwise healthy individuals. Key learning points include the necessity of early recognition and intervention to mitigate severe complications and the potential need for thromboprophylaxis in varicella pneumonia patients and suggests the potential need for thromboprophylaxis in patients with varicella pneumonia to prevent thrombotic events.

## Introduction

Varicella-zoster virus (VZV) is a ubiquitous *Human alphaherpesvirus* that causes varicella (chickenpox) and herpes zoster (shingles). 1 Varicella (chickenpox) is a common infection of childhood, typically affecting children aged 2 to 8 years and usually follows a benign course. 2 VZV pneumonia (VZV-P) can occur as a complication, particularly in adults, with more men than women affected, with a male-to-female ratio or 3:1 and a mean age of 31 years (range: 20–44 years). 3 It can cause pulmonary complications that may be mild initially but could progress to respiratory failure and cause acute respiratory distress syndrome (ARDS). 4

VZV-P is a potentially fatal condition that mainly affects adults and immunocompromised individuals. The incidence of VZV-P in healthy adults varies between 0.3 and 50%, with a reported mortality rate ranging from 2.15 to 20% in the general population and up to 41% in pregnant women. 5 In the literature, only a few cases have been reported linking varicella infection to thrombosis, and these often involve additional predisposing factors such as genetic mutations. For instance, in this case report, the patient had a factor V Leiden mutation and activated protein C resistance, which are known genetic predispositions to thrombosis. High-resolution computed tomography (CT) scans show multiple bilateral nodules without a surrounding halo of ground-glass attenuation. 6 Acyclovir is the first-line treatment for severe or complicated varicella infections, particularly in immunosuppressed patients and can reduce the duration and severity of complications, particularly when given within 24 hours of the onset of symptoms. 7

The co-occurrence thromboembolic disease (TED) with a severe presentation of VZV-P underscores the necessity for health care providers to consider a broader differential diagnosis when encountering varicella patients with severe pneumonia. The identification of genetic predispositions to thrombosis in these patients can lead to more tailored and effective management strategies. Moreover, it points to the importance of varicella vaccination, especially in individuals known to have a higher risk of thrombotic events.

## Case Report

A young patient with no previous medical problems presented to the emergency department with a 5-day history of fever and diffuse rash, initially vesicular and then evolving into vesiculopustular lesions. His symptoms included fatigue, anorexia, and joint pain, followed by the abrupt onset of dyspnea and dry cough on the fifth day. The patient's past medical history revealed Crohn's disease diagnosed at the age of 15 years, which was well controlled without medication for the past decade, and this predisposes to venous thromboembolism (VTE)/pulmonary embolism (PE), so he should have been on prophylaxis. The patient had no other risk factors for severe VZV or TED; in particular, there was no history of recent travel, immunosuppressives, or prothrombotic disorders. On examination, his vital signs on admission were as follows: blood pressure 110/80 mm Hg, heart rate 120 bpm, oxygen saturation 82% on room air, temperature 39°C, and respiratory rate 22 breaths per minute. Auscultation revealed fine crackles and wheezing in the chest, while the skin showed numerous papules with an erythematous macular base, several vesicles filled with thick fluid, and some erosions. All other physical examination findings were unremarkable.

**Fig. 1:**
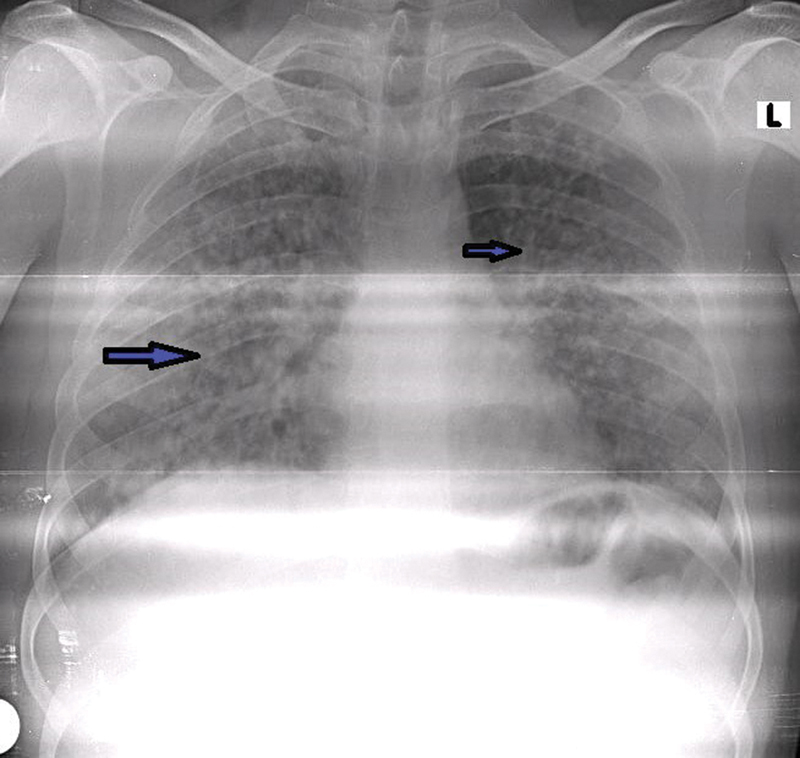
Chest X-ray showed diffuse nodular infiltration in all lobes. Arrows highlight regions of diffuse nodular infiltration in all lung lobes on the chest X-ray.

**Fig. 2:**
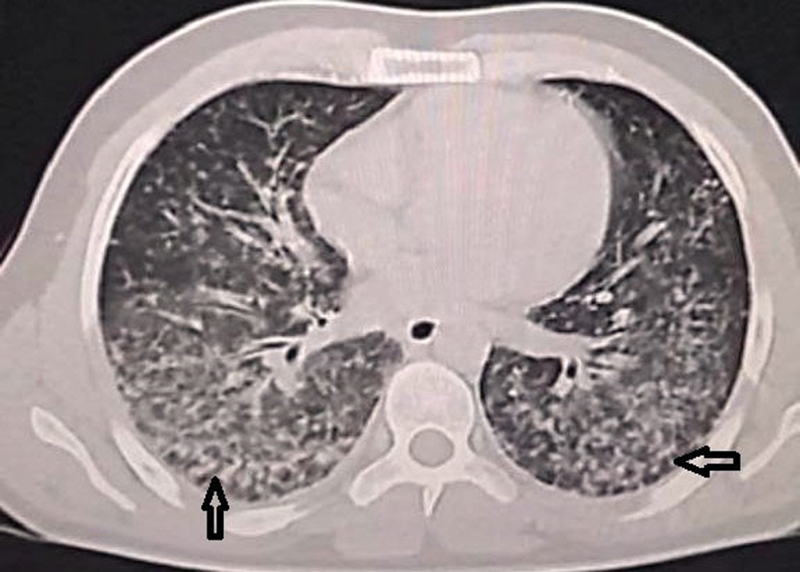
Thoracic computed tomography (CT) showed diffuse infiltration with bronchogram. Arrows point to the miliary nodular pattern and tree-in-bud sign on thoracic CT.

Laboratory tests revealed the following: white blood cell (WBC) 8,300/mm ^3^ (70% neutrophils), Htc 40.6%, plt 277,000/mm ^3^ , sedimentation 12 mm/h, C-reactive protein (CRP) 7.6 mg/dL (<0.8), international normalized ratio (INR) 1, prothrombin time (PT) 15, ALT 51 IU/L, creatinine 1, procalcitonin 0.16, HBS Ag, and anti-HCV negative ( Table 1 ). Chest X-ray showed diffuse pulmonary nodular consolidation ( Fig. 1 ), and thoracic CT showed a diffuse miliary nodular pattern and tree-in-bud sign ( Fig. 2 ).

**Table 1:** Laboratory examination result

Laboratory analysis	Value
White blood count (103/μL)	8,300
Neutrophil (%)	70
Lymphocytes (%)	26
Monocytes (%)	4
Hemoglobin (g/dL)	13.1
C-reactive protein	13
Prothrombin time	14
International normalized ratio	1
Erythrocyte sedimentation rate	12
Procalcitonin	0.16

He was admitted to the intensive care unit due to his acute, type 1 respiratory failure and was administered continuous oxygen via a mask and a nonrebreathing bag at 12 L/min. The treatment regimen included intravenous (IV) acyclovir (10 mg/kg three times a day), prednisolone (0.5 mg/kg/d), prophylactic heparin, and physical therapy. Prophylactic heparin was given because the patient was unable to ambulate for the first 24 hours, increasing the risk of TED. The decision to use prophylactic heparin was based on the standard practice of preventing VTE in immobilized patients, particularly in the context of severe infection and systemic inflammation, which are known to elevate the risk of clot formation. Within a day, the patient's condition improved, and he was transitioned to a simple mask at 6 L/min, followed by a nasal cannula at 2 L/min. By the third day, his improved respiratory status allowed for his transfer to a general ward, as supplemental oxygen was no longer required.

**Fig. 3:**
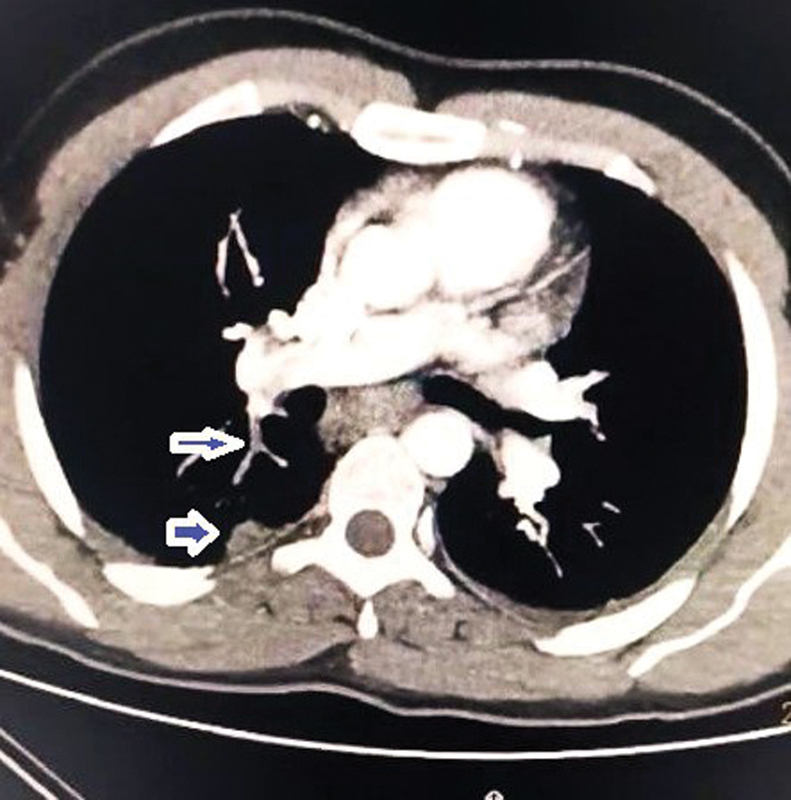
Thoracic computed tomography (CT) angiography revealed thrombus in the branches of the right pulmonary artery and pulmonary infarction. Arrows mark thrombosis in branches of the right pulmonary artery and a pulmonary infarction.

Four days after his initial discharge, the patient was readmitted due to swelling and pain in his left leg with right-sided chest pain. Doppler ultrasound showed a deep venous thrombosis (DVT) in the left superficial femoral and popliteal veins, while a thoracic CT angiogram revealed extensive thrombosis in the right pulmonary arteries and a pulmonary infarction ( Fig. 3 ). He was commenced on a treatment dose of low-molecular-weight heparin at 1 mg/kg/12 h subcutaneously.

Ethical approval was not required for this case report at our institution, Faculty of Medicine at Aleppo University, Aleppo, Syria.

## Discussion

Varicella, caused by VZV, usually has a benign course in healthy individuals. However, severe complications may arise, especially in adult populations.

Pneumonia is the most common and the most serious complication of VZV infection in healthy adults. 8 VZV-P induces tachypnea, chest tightness, cough, dyspnea, fever, pleuritic chest pain, and hemoptysis. 9 VZV-P usually develops within 1 to 6 days of the onset of varicella. 10 In a case series on the complications of chickenpox in 102 patients, 28.4% patients developed VZV-P; all the patients, except 2, were males. 11 In our patient, other than his well-controlled (without medication) Crohn's disease, there were no other known risk factors for TED; in addition, he received prophylaxis for the duration of his initial stay. In other instances, genetic predispositions such as factor V Leiden mutation and activated protein C resistance significantly increase the risk of thrombotic complications. Investigating these factors in patients with unusual thrombotic presentations following VZV infection is crucial for understanding the underlying mechanisms and improving patient management. However, these are unavailable in Syria to which this patient presented.

Upon admission, diffuse miliary nodular consolidation was observed on the chest X-ray ( Fig. 1 ). Computed tomography was subsequently performed to further evaluate the lungs, revealing a random nodular pattern predominantly affecting the lower lobes ( Fig. 2 ). Based on clinical symptoms and radiological findings, a diagnosis of varicella pneumonia was made. Treatment consisted of oxygen therapy via mask and nonrebreathing bag at 12 L/min, IV fluids at a rate of 50 mL/h, acyclovir at a dose of 10 mL/kg every 8 hours, prophylactic proton pump inhibitors, prednisolone at a dose of 0.5 mg/mg acetaminophen, and prophylactic heparin.

Otherwise healthy adults and particularly immunocompromised patients who contract varicella should be treated with antiviral medications. The approved antiviral drugs for varicella include acyclovir, valaciclovir, and famciclovir, with valaciclovir and famciclovir being favored for their high bioavailability; the treatment typically lasts for 7 to 10 days and should be customized based on the patient's clinical condition. 12 In severe cases, IV acyclovir at 10 mg/kg three times a day should be used with careful hydration and fluid management to avoid renal impairment.

Varicella pneumonia is a rare complication in nonimmunosuppressed patients. However, the occurrence of this condition along with thromboembolic events, such as DVT and PE, raises important questions. Although the patient showed early mobility and rapid symptom improvement, the relationship between varicella infection and thromboembolic events remains under investigation. It is hypothesized that varicella infection may lead to a hypercoagulable state due to its systemic inflammatory response. Viral-induced hypercoagulability (VZV infection) has been linked to elevated levels of procoagulant factors and reduced levels of anticoagulant proteins. VTE associated with VZV is often due to the production of antiphospholipid antibodies. Additionally, primary thrombophilic conditions arise from qualitative or quantitative deficiencies in antithrombotic proteins. 13 Additionally, varicella-induced endothelial damage and prothrombotic effects may heighten the risk of thromboembolic complications. Infection can provoke a strong systemic inflammatory response, characterized by the release of proinflammatory cytokines. These cytokines can activate the coagulation cascade, increasing the risk of thromboembolic events. Elevated levels of interleukin-8 (IL-8), IL-6, and matrix metalloproteinase-2 (MMP-2) can contribute to inflammation and vascular wall damage, which are key features of VZV vasculopathy. 14 This case highlights the importance of monitoring thromboembolic events in varicella patients, regardless of preexisting risk factors. Further research is needed to understand the mechanisms underlying these atypical complications and to establish evidence-based management guidelines. Health care providers should be vigilant for potential thromboembolic complications in varicella-infected individuals, especially in cases that deviate from the expected disease course.

Such cases underscore the importance of considering a broader range of diagnostic possibilities when treating varicella patients exhibiting unusual symptoms. Identifying genetic predispositions to thrombosis can lead to more precise and effective management strategies. Furthermore, this case highlights the critical role of varicella vaccination, especially for individuals at higher risk of thrombotic events. By documenting and analyzing these rare cases, the medical community can enhance its understanding of the potential complications associated with common viral infections, thereby improving preventative and therapeutic approaches.

## Conclusions

Varicella pneumonia, when coupled with VZV infection, can lead to rare thrombotic complications. Early treatment with IV acyclovir and oxygen supplementation significantly reduced pulmonary complications in this case. This case emphasizing the need to consider varicella as a potential cause of thromboembolic events, even in otherwise healthy individuals highlighting the critical role of timely recognition and intervention in optimizing patient outcomes. Further research is needed to understand the underlying mechanisms and risk factors associated with these rare varicella-related complications.
